# A closer look at *Aspergillus*: online monitoring via scattered light enables reproducible phenotyping

**DOI:** 10.1186/s40694-019-0073-x

**Published:** 2019-08-05

**Authors:** Roman P. Jansen, Carina Beuck, Matthias Moch, Bianca Klein, Kira Küsters, Holger Morschett, Wolfgang Wiechert, Marco Oldiges

**Affiliations:** 1 Forschungszentrum Jülich, Institute of Bio- and Geosciences-Biotechnology (IBG-1), Jülich, Germany; 2 Computational Systems Biotechnology (AVT.CSB), RWTH Aachen University, Aachen, Germany; 3 Institute of Biotechnology, RWTH Aachen University, Aachen, Germany

**Keywords:** *Aspergillus giganteus*, Microtiter plate, Miniaturized cultivation, Antifungal protein, High throughput bioprocess development, Morphology engineering, Online monitoring

## Abstract

**Background:**

Filamentously growing microorganisms offer unique advantages for biotechnological processes, such as extraordinary secretion capacities, going along with multiple obstacles due to their complex morphology. However, limited experimental throughput in bioprocess development still hampers taking advantage of their full potential. Miniaturization and automation are powerful tools to accelerate bioprocess development, but so far the application of such technologies has mainly been focused on non-filamentous systems. During cultivation, filamentous fungi can undergo remarkable morphological changes, creating challenging cultivation conditions. Depending on the process and product, only one specific state of morphology may be advantageous to achieve e.g. optimal productivity or yield. Different approaches to control morphology have been investigated, such as microparticle enhanced cultivation. However, the addition of solid microparticles impedes the optical measurements typically used by microbioreactor systems and thus alternatives are needed.

**Results:**

*Aspergillus giganteus* IfGB 0902 was used as a model system to develop a time-efficient and robust workflow allowing microscale cultivation with increased throughput. The effect of microtiter plate geometry, shaking frequency and medium additives (talc and calcium chloride) on homogeneity of culture morphology as well as reproducibility were analyzed via online biomass measurement, microscopic imaging and cell dry weight. While addition of talc severely affected online measurements, 2% (w v^−1^) calcium chloride was successfully applied to obtain a highly reproducible growth behavior with homogenous morphology. Furthermore, the influence of small amounts of complex components was investigated for the applied model strain. By correlation to cell dry weight, it could be shown that optical measurements are a suitable signal for biomass concentration. However, each correlation is only applicable for a specific set of cultivation parameters. These optimized conditions were used in micro as well as lab-scale bioreactor cultivation in order to verify the reproducibility and scalability of the setup.

**Conclusion:**

A robust workflow for *A. giganteus* was developed, allowing for reproducible microscale cultivation with online monitoring, where calcium chloride is an useful alternative to microparticle enhanced cultivation in order to control the morphology. Independent of the cultivation volume, comparable phenotypes were observed in microtiter plates and in lab-scale bioreactor.

**Electronic supplementary material:**

The online version of this article (10.1186/s40694-019-0073-x) contains supplementary material, which is available to authorized users.

## Background

Filamentous microorganisms have always played an important role in industrial biotechnology: Citric acid production with *Aspergillus niger* dates back to 1919 in Belgium [1] and since then *Aspergillus* sp. have been established as industrial workhorses. Filamentous fungi and especially the *Aspergillus* species offer unique advantages such as an outstanding secretory capacity. 27% of commercial enzyme production, such as amylases, catalases and proteases, is performed utilizing *Aspergillus* [2]. Besides their performance as a secretory enzyme production host, filamentous fungi are also attractive candidates for secondary metabolite production as 38% of all known bioactive metabolites are originating from filamentous fungi [3]. Since a steadily increasing number of genomes become available, an increase in their utilization as a source for novel enzymes and metabolites is expected [4].

However, due to their complex morphology, the development of industrial processes using filamentous fungi remains quite challenging. Analogous to bacterial cultivations, the submerged cultivation is state of the art in industry [5]. In submerged culture, the mycelia can either be freely dispersed throughout the liquid volume or form macroscopic aggregates. If such aggregates form rather loose structures, they are referred to as mycelial clumps, whereas more compact and dense structures are called pellets [6]. The different morphology has a great impact on both productivity and physical properties of the cultivation suspension. At high biomass concentration loose mycelia and mycelial clumps tend to come in contact with each other, making the suspension highly viscous and inhomogeneous [6]. On the other hand, a diffusion limitation might occur within larger pellet structures which might reduce productivity or even result in cell death and lysis in the inner pellet volume [5]. Nevertheless, the suitable morphology has to be identified for each strain and process individually.

Many process parameters have been described as possible factors for morphology engineering. Osmolality, pH, power input and initial spore concentration can be adjusted to control the desired morphology [7, 8]. Furthermore, in the past years a novel approach for morphology control, the so-called microparticle enhanced cultivation (MPEC), has been developed. Through addition of insoluble microparticles, the pellet size can be decreased [9, 10]. This technology has been successfully applied for multiple organisms, such as *A. niger* [11, 12] and *A. terreus* [13].

To establish a production process many preliminary experiments need to be conducted identifying the best performing strain and optimal process parameters. To accelerate this laborious task an adequate cultivation throughput is necessary. Microtiter plates (MTPs) are a well-established tool for such screening campaigns [14]. As a consequence, multiple studies investigating suitable MTP cultivation conditions for various filamentous fungi have been carried out. However, most often these experiments used end point measurements only to receive process information [15–18]. Comparatively, bacterial phenotyping systems often utilize microbioreactor systems with non-invasive online measurement of pH, dissolved oxygen and biomass via optical methods [14, 19]. Such systems allow for better process control and insight without reducing the experimental throughput. Until now, such systems have only been applied for filamentous bacteria [20] or without the use of online measurement for filamentous fungi [21].

In this study, a cultivation strategy was developed to enable online monitored microbioreactor cultivation for *Aspergillus*. Well geometry, power input, medium composition and influence of additives were addressed to ensure reproducible cultivation. The production of an antifungal protein (AFP) by *A.* *giganteus* was analyzed and the optimized conditions were tested regarding scalability into a lab-scale stirred tank bioreactor. AFP is a well characterized protein that exclusively inhibits the growth of several fungi such as strains of *Fusarium* and *Aspergillus* making it an interesting candidate for biotechnological application [22–24].

## Results and discussion

### Microbioreactor reference cultivation

**Fig. 1:**
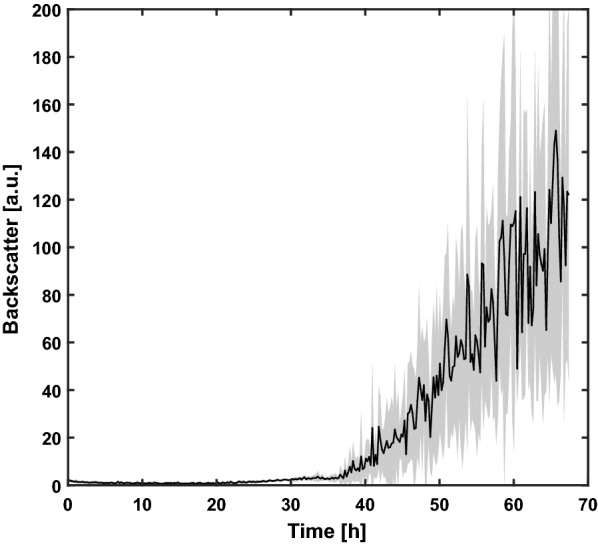
A BioLector reference cultivation. Complex yeast extract peptone dextrose medium was inoculated with 2 × 10^6^ spores mL^−1^. *Aspergillus giganteus* was cultivated in a Flowerplate at 1100 rpm and 30 °C for 70 h. The biomass was analyzed non-invasively via scattered light measurement. The mean (thick line) and standard deviation (lighter area) of eight biological replicates are shown

As a starting point, standard conditions for bacterial microbioreactor cultivation were applied to *Aspergillus giganteus* in the BioLector setup. A Flowerplate, which is a specialized microtiter plate optimized towards high oxygen transfer rate, allowing scattered light measurement, was inoculated with 2 × 10^6^ spores mL^−1^ and incubated at 1100 rpm and 30 °C. The non-invasive backscatter measurement of eight biological replicates was compared as a measure for reproducibility (Fig. 1). Similar to optical density the backscatter signal can provide information about biomass concentration. The mean of all replicates is represented as a thick line and the standard deviation as the corresponding light shadow surrounding it. Due to direct inoculation with spores and necessary time for germination, no increase in backscatter measurement is distinguishable within the first 20 h of cultivation, in contrast to inoculation with a starter culture (Fig. 1). After 37 h, the *Aspergillus* cell structures reach the measurement threshold and an increase in backscatter can be detected throughout the cultivation process. However, compared to BioLector cultivations with other microorganisms [20, 25, 26], a highly variable and nonstable backscatter increase can be observed. Furthermore, large standard deviations of the scattered light signal indicate poor reproducibility of the growth and the optical measurement itself (Additional file 1). The complex morphology of *Aspergillus* with varying pellet sizes and densities might hinder the consistent measurement of the true backscatter signal. Consequently, parameters such as well geometry, shaking frequency or addition of morphology controlling agents need to be addressed to achieve reproducible conditions for cultivation and backscatter-assisted biomass monitoring.

### Influence of morphology controlling agents on scattered light biomass measurement

**Fig. 2:**
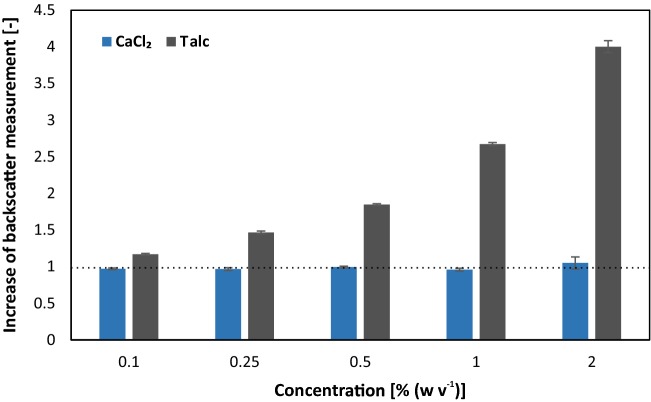
Influence of talc and calcium chloride on the backscatter measurement performed in the BioLector. Different concentrations of talc and calcium chloride were added to YEPD medium and transferred into a Flowerplate. The backscatter measurement was performed in the BioLector in triplicates and the mean including standard deviation are shown. The dotted line represents the reference backscatter measurement of YEPD medium without additives

MPEC is a popular technique in order to control filamentous morphology, where talc is one of the commonly used additives. Due to the low solubility of talc microparticles, the first step was to determine the basic influence of different talc concentrations on the backscatter measurement of the BioLector (Fig. 2), without any filamentous fungi present. With increasing talc concentration added to the complex yeast extract peptone dextrose (YEPD) medium, the backscatter signal increases strongly. A series of talc concentrations ranging from 0.1 to 2% (w v^−1^) was investigated. The lowest talc concentration of 0.1% (w v^−1^) already showed a significant increase of backscatter value and 0.5% (w v^−1^) talc resulted in a doubled backscatter value, making biomass measurement impossible and clearly limiting its applicability in this context. As an alternative additive influencing the morphology of filamentous microorganisms calcium chloride can be used [27, 28]. In contrast to talc, calcium chloride is soluble up to high concentrations above 700 g L^−1^ in aqueous solutions at physiological temperature. In the same concentration range from 0.1% (w v^−1^) up to 2% (w v^−1^) calcium chloride showed no impact on the backscatter signal. As a consequence the concentration of additives for the following cultivation experiments was set to 0.1% (w v^−1^) for talc and 2% (w v^−1^) for calcium chloride.

### Influence of plate geometry, shaking frequency and morphology-regulating additives on morphology and reproducibility

In order to determine the cultivation setup for *A. giganteus* that provides the highest reproducibility, the influence of plate geometry, shaking frequency and medium additives was tested. Two types of microtiter plate well geometries were investigated, i.e. round well plates and flower shaped plates. Due to the distinctive geometry of the Flowerplate, it is similar to baffled shake flasks, whereas on the other hand, a round well plate has a similar geometry to an unbaffled shake flask. Besides the well geometry the shaking frequency is a key parameter to influence the energy dissipation into the culture broth. Higher shear forces at increasing rotation speeds can be observed and influence *Aspergillus’* morphology. The effect was analyzed for 600, 850 and 1100 rpm in both Flowerplates and round well plates. Moreover, the impact of both CaCl_2_ and talc as morphology controlling agents was determined at concentrations of 2% (w v^−1^) and 0.1% (w v^−1^), respectively.

The backscatter measurement of eight biological replicates for each condition was analyzed as a measure of reproducibility (Additional files 2 and 3). The relative coefficient of variation was calculated for each measurement point. As an overall characteristic of reproducibility, relative mean coefficient of variation (rmc_v_) (Eq. 2) was used for each tested condition (Table 1). In general the use of CaCl_2_ was superior to talc at the applied concentration: For all shaking frequencies and both well geometries, the rmc_v_ is comparably low in the range of 3–22% when CaCl_2_ was used. Moreover, the impact of CaCl_2_ on the reproducibility of the backscatter signal seems to be higher compared to influences by well geometry and shaking frequency. The concentration of 0.1% (w v^−1^) talc seems to be too low to provide beneficial effect of MPEC to create homogenous and reproducible cultivation conditions.

**Table 1:** Calculated relative mean coefficient of variation (rmc_v_) from online biomass measurement for cultivation in either a round well plate (RWP) or a Flowerplate (FP) at different shaking frequencies

rmc_v_	600 rpm		850 rpm		1100 rpm	
	FP	RWP	FP	RWP	FP	RWP
YEPD medium	0.20	0.30	0.38	0.16	0.62	0.18
+ CaCl_2_	0.09	0.05	0.22	0.03	0.19	0.03
+ talc	0.91	0.36	0.68	0.18	0.68	0.18

**Fig. 3:**
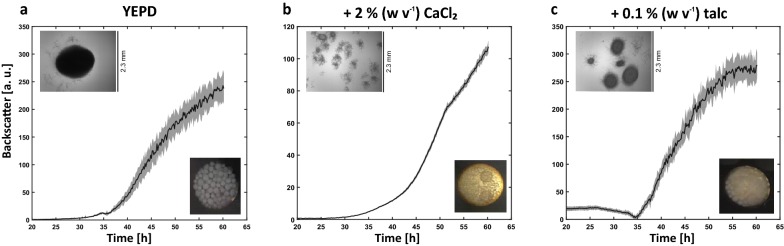
Microtiter plate cultivation of *A. giganteus* with quasi-continuous online measurement of biomass via backscatter. Eight biological replicates were cultivated at 850 rpm in a round well plate. The mean (thick line) with standard deviation (grey area) was plotted over the cultivation duration of 60 h. YEPD reference medium (**a**) was compared against addition of CaCl_2_ (**b**) and talc (**c**). Microscopic pictures (×40) and pictures from the bottom of a well were taken to analyze pellet formation were taken at the end of the cultivation after 60 h. Each image shows a clipping of 2.85 mm in width and 2.3 mm in height

The cultivation data for round well plate cultivation with 850 rpm is shown in Fig. 3. To illustrate the different effects on the culture morphology this condition is taken as a representative example. The backscatter measurement, microscopic images and pictures from the bottom of the well demonstrate the impact of CaCl_2_ (Fig. 3b) and talc (Fig. 3c) in comparison to YEPD reference medium (Fig. 3a).

The cultivation in complex YEPD medium resulted in pellets with a diameter of up to 2 mm with an rmc_v_ of 16%. This morphology has a negative impact on the backscatter signal leading to an inconsistent measurement and consequently high signal to noise ratio, depending on the current location of the pellets during the measurement (Fig. 3a). The addition of either CaCl_2_ or talc decreases pellet size as can be seen in the microscopic images. The culture treated with talc forms smaller pellets with varying diameter, from 0.2 to 1 mm. Nevertheless, this seems to impact the backscatter measurement and a substantial standard deviation and therefore an rmc_v_ of 18% are obtained. Furthermore, due to the varying pellet sizes, the diffusion of nutrients into the pelleted structure most likely will differ as well, potentially inducing process inhomogeneity. The addition of CaCl_2_ induces the most pronounced change in morphology. Mycelial aggregates with a diameter of 0.2 mm can be seen via microscope imaging. The backscatter measurement of eight replicates leads to very reproducible readings (low signal noise) and finally to a very small rmc_v_ of 3% (Fig. 3b). The low impact of talc microparticles and the beneficial one of CaCl_2_ on the noise of scattered light measurements was similarly observed for all tested well geometries and shaking frequencies. The observed influence of this medium additive seems to be much stronger on the rmc_v_ compared to the physical power input varied through changes in shaking frequencies and well geometry. Increasing shaking frequency above 850 rpm resulted in a slight decrease in pellet size only, but increased wall growth effects at the same time (data not shown). In literature, some studies report an effect of calcium chloride on signaling pathways, such as calcineurin-dependent calcium signaling in filamentous fungi: Calcium signaling might impact conidia germination, hyphal growth regulation and cell morphogenesis [29–32]. This might also effectuate changes on macromorphology.

### Development of an optimized culture medium

**Fig. 4:**
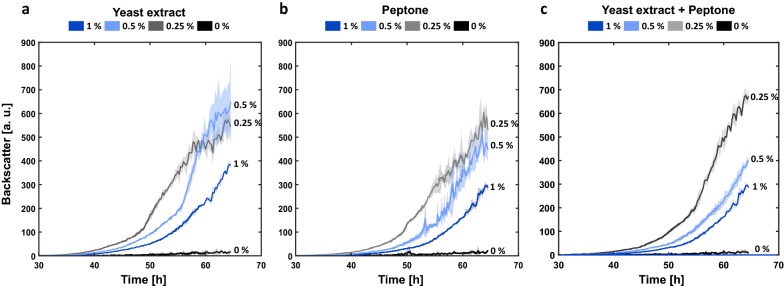
Microtiter plate cultivation of *A. giganteus* with medium based on Sinha. To improve growth different complex components were supplemented with different concentrations ranging up to 1% (w v^−1^). Yeast extract (**a**), peptone (**b**) and a combination of both (**c**) was tested. The influence of each component on growth was analyzed regarding its impact on the backscatter measurement. The cultivation was performed at 30 °C and 850 rpm in a round well plate with addition of 2% (w v^−1^) calcium chloride

As batch to batch variation of complex media components may result in reduced process robustness, defined media are often favorable. However, for filamentous fungi it is stated that a small amount of complex medium components supports growth and induces filamentous morphology during cultivation [16]. As a consequence the impact of different complex components such as yeast extract, peptone or a combination of both was tested on the growth and reproducibility of the backscatter signal of *A. giganteus* in microbioreactor setup with 2% (w v^−1^) CaCl_2_ for morphology control (Fig. 4). Different concentrations ranging from 0.25 to 1% (w v^−1^) were added to a defined medium based on Sinha [33]. The growth of the filamentous fungi based on the backscatter increased for all added concentrations in comparison to the negative control without complex components. This finding suggests spore germination is only induced in the investigated scenario, if complex components are provided and apparently, an optimum is reached at 0.25 (w v^−1^) concentration.

With respect to the standard deviation of the backscatter signal, the most reproducible cultivation condition was 0.25% (w v^−1^) of yeast extract and 0.25% (w v^−1^) of peptone (Fig. 4c). It can be expected that yeast extract and peptone differ in their specific composition of nutrients and it seems that there is an added value when used in combination. A higher concentration of complex supplements did not result in a further improvement on scattered light reproducibility and already lead to a reduction in the growth of *A. giganteus* as can be seen by reduced growth performance, which might be due to some inhibitory effects.

The scattered light measurement utilized by the BioLector system is an optical measurement and as such it may strongly depend on the morphology, i.e. it does not necessarily show linear correlation with cell dry weight. However, cell dry weight is usually not impacted by altering morphology and therefore serves as a good offline reference for validation of biomass measurement techniques.

**Fig. 5:**
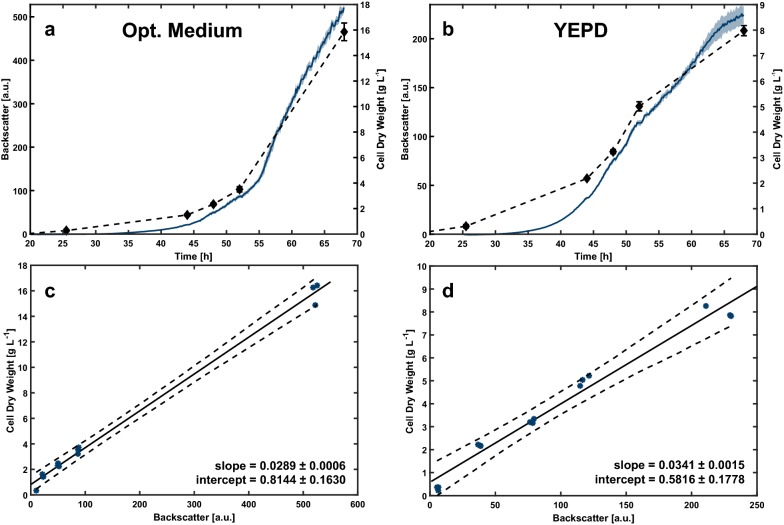
MTP cultivation of *A. giganteus* in both optimized medium (**a**) and complex YEPD medium (**b**) with 2% (w v^−1^) calcium chloride. The cultivation was performed at 30° C and 850 rpm in a round well plate. The mean (thick line) and standard deviation of the backscatter measurement of three biological replicates are shown. Three biological replicates of each condition were sampled at four different time points to determine cell dry weight. **c** (opt. Medium) and **d** (YEPD) show a linear correlation between backscatter and cell dry weight for each medium along process runtime. Each of the 15 sampled wells was used for the correlation where the offline measured cell dry weight was plotted against its specific backscatter value prior to the sampling time point. The dotted lines show the 95% confidence intervals for each regression

The backscatter signals for cultivation in optimized medium with CaCl_2_ result in much higher values up to 500 a.u. and also higher cell dry weight up to 16 g L^−1^ in comparison to YEPD medium with 200 a.u. and 8 g L^−1^. Regarding the individual conditions the offline measured cell dry weight follows the trend of the respective backscatter signal quite well (Fig. 5a, b). Even though the morphology of the filamentous fungi changes from spores to large pellets over the duration of cultivation, orthogonal distance regression suggests a linear relationship between the optical scattered light measurement and cell dry weight (Fig. 5c, d). Thus, under the experimental conditions applied, scattered light represents a valid measure for biomass along process runtime. However comparing both regressions, the individual slopes differ significantly (p < 0.05). Consequently, such correlations seem rather specific and may not be universally applied. They have to be re-assessed when medium composition or process parameters are changed.

### Bioreactor cultivation

**Fig. 6:**
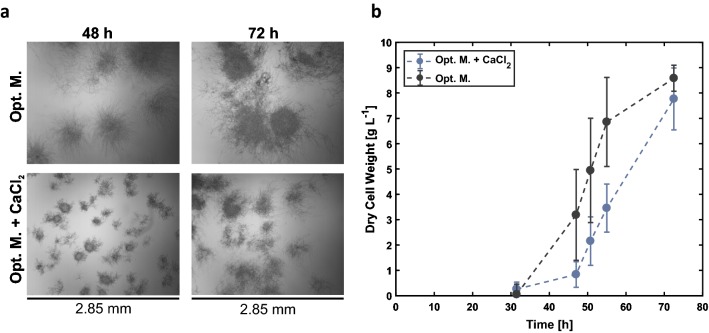
Bioreactor cultivation of *A. giganteus* with the optimized medium. **a** Microscopic images (×40) of pellets at different time points for both conditions. **b** Cell dry weight (g L^−1^) of samples (n = 3) taken throughout the bioreactor cultivation. The influence of calcium chloride can be seen in the reduced pellet size compared to untreated pellets. Each image shows a clipping of 2.85 mm in width and 2.3 mm in height

Microtiter plate cultivation is an ideal system for preliminary screening experiments. Nevertheless, it is essential to perform subsequent experiments in stirred tank bioreactors to validate the results. Hence, calcium chloride supplementation was applied in lab-scale bioreactor experiments and led to a strong reduction of pellet size throughout the whole cultivation (Fig. 6a), which is fully comparable to results observed in the microtiter plate. In comparison to the MTP cultivation, no obvious difference in pellet size is observed, when cultivated with CaCl_2_ addition (Additional file 4). Although varying geometry and power input, i.e. stirred not shaken, a similar effect of CaCl_2_ addition compared to the BioLector cultivation can be proven, showing that the pellet size is strongly reduced throughout the cultivation without affecting the overall biomass production. Due to a lack of bioreactor cultivation data of *A. giganteus* in literature, it seems that this is the first report of such data for *A. giganteus* from a controlled bioreactor cultivation environment.

### Comparative supernatant analysis of cultivation scales

**Fig. 7:**
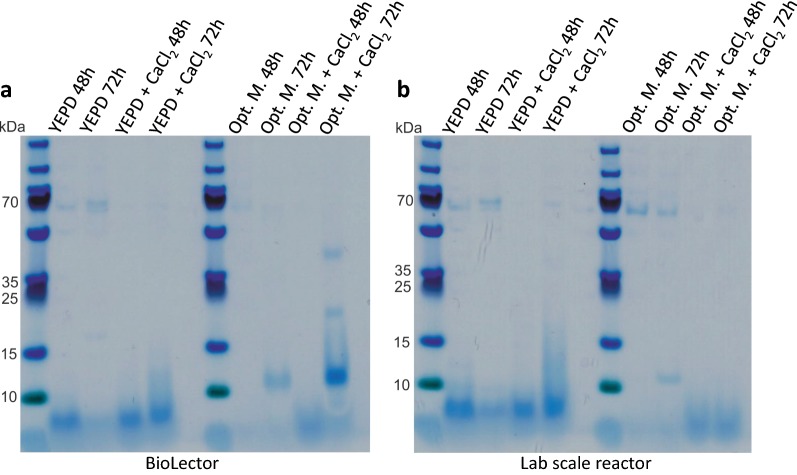
SDS-PAGE gel of samples with different cultivation conditions. Samples of each process condition were loaded for two different time points (1 = 48 h and 2 = 72 h) onto the gel. The BioLector supernatant (**a**) is highly comparable to the supernatant of cultivation in a lab-scale bioreactor (**b**). AFP has a molecular weight of 6 kDA and shows the most prominent bands at the bottom of the gel

The proteins secreted in the supernatant of both microtiter plate and bioreactor cultivations were analyzed via SDS-PAGE and visualized via Coomassie brilliant blue staining to compare transferability between the two systems. It is reported that *A. giganteus* secrets two small highly basic proteins, AFP with an molecular weight of 6 kDa and α-sarcin with a size of 17 kDa [34]. The stained gel shows the most prominent bands at the bottom below the 15 kDa marker (Fig. 7). Both AFP and α-sarcin were identified unambiguously via LC-MS/MS by their peptide fingerprints of tryptic digest and AFP and α-sarcin gene sequence obtained from NCBI. In general, a very similar protein pattern can be detected comparing both cultivation systems. Moreover, a slightly darker band and consequently an increased protein concentration can be seen for the majority of cases where CaCl_2_ was added to the medium. Especially comparing the cultivations with the optimized medium, the gel staining suggests an increase of AFP secretion, when CaCl_2_ was added. The analyzed protein pattern in the supernatant for both systems is comparable regarding AFP production, indicating a good scalability between BioLector and lab-scale reactor. Only the α-sarcin band seen in the BioLector cultivation with optimized medium and calcium chloride supplementation can not be detected in the supernatant of the lab-scale reactor cultivation. For a detailed product quantification a downstream purification could be performed as described in literature previously [35].

## Conclusions

For accelerated bioprocess development, miniaturization and parallelization are valuable tools. Microbioreactor cultivations offer increased throughput and an online biomass signal while simultaneously reducing manual workload and handling error potential. However, most filamentous fungi are screened either in shake flasks or normal MTPs lacking relevant bioprocess information. So far, no studies on *Aspergillus* cultivation with online biomass monitoring have been reported and the application of morphology engineering technologies such as MPEC will impede the online measurement. In this study cultivation strategies were developed allowing tracking of cultivation progress by optical online measurements. Despite changing morphology during process runtime, optical measurements could be linearly correlated to cell dry weight for a given and constant set of process conditions. Highly reproducible cultivations for biological replicates were performed with an rmc_v_ as low as 4%. However, no absolute quantification of online biomass can be performed, when different media and cultivation conditions are used, since each condition results in a specific biomass backscatter correlation. Moreover, sacrifice sampling has to be performed for offline measurements, which is compensated by increased cultivation throughput. Addition of CaCl_2_ has been proven to be a good alternative to MPEC in order to control the filamentous morphology. It strongly decreases the pellet size up to ten fold without reducing overall biomass production. The effect is rather independent from power input and can be reproduced in stirred tank bioreactors. Furthermore, the analysis of the supernatant for both systems was very similar, indicating good transferability. The influence of multiple complex components on the growth of *A. giganteus* was analyzed. As a next step, integration into automated process development platforms would be beneficial to further accelerate optimization of bioprocesses using filamentous fungi. Standardized bacterial workflows need to be adjusted and novel ones, such as automated morphology analysis, need to be developed.

## Materials and methods

### Chemicals

All chemicals (analytical grade) were purchased from Carl Roth (Karlsruhe/Germany) or Sigma-Aldrich (St. Louis, Missouri/USA).

### Microbial strains, medium and spore production

The *Aspergillus giganteus* strain IfGB 0902 was kindly provided by the Department of Applied and Molecular Microbiology of the Technical University of Berlin. 10 µL of the spore suspension was plated on potato dextrose agar plates and incubated at 37 °C in an incubation chamber for 7 days. After scraping the spores from the agar plate through addition of a 20% (v v^−1^) glycerol in 0.9% (w v^−1^) NaCl solution, the mixture was filtrated and the spores were counted utilizing an improved Neubauer chamber (VWR, Radner, Pennsylvania, USA). The spores were diluted with a 20% (v v^−1^) glycerol in 0.9% (w v^−1^) NaCl solution to a final concentration of 1 × 10^8^ spores mL^−1^, aliquoted and stored at − 80 °C. A complex YEPD medium consisting of 10 g L^−1^ yeast extract, 20 g L^−1^ dextrose and 20 g L^−1^ peptone in water with the pH adjusted to 3.5 was used. A defined medium described in Sinha et al. [33] was slightly adapted, consisting of nitrate salts (0.52 g L^−1^ KCl, 1.52 g L^−1^ KH_2_PO_4_, 6 g L^−1^ NaNO_3_, 0.52 g L^−1^ MgSO_4_ * 7 H_2_O), trace elements (50 mg L^−1^ Na_4_EDTA, 2.25 mg L^−1^ ZnSO_4_ * 7 H_2_O, 11 mg L^−1^ H_3_BO_3_, 5 mg L^−1^ FeSO_4_ * 7 H_2_O, 5 mg L^−1^ MnCl_2_ * 4 H_2_O, 1.7 mg L^−1^ CoCl_2_ * 6 H_2_O, 1.6 mg L^−1^ CuSO_4_ * 5 H_2_O, 0.085 mg L^−1^ Na_2_MoO_4_* 2 H_2_O) and vitamin solution (1 mg L^−1^ of each biotin, pyridoxine, thiamine, riboflavin, *p*-aminobenzoic acid and nicotinic acid) and 80 g L^−1^ glucose with the pH set to 3.5. The stocks were prepared and sterilized separately and added immediately before the cultivation. The vitamin and trace element stocks were aliquoted and stored at − 20 °C to ensure identical conditions for each experiment.

### Microbioreactor cultivation

Cultivation was performed utilizing a BioLector device. 48-Well round well plates (MTP-R48-BO) and Flowerplates (MTP-48-BO) sealed with an evaporation reducing gas-permeable sealing foil (F-GPR-48-10) were used (all m2p-labs GmbH, Baesweiler, Germany). The medium was inoculated to a concentration of 2 × 10^6^ spores mL^−1^. The incubation chamber of the BioLector was set to 30 °C and ≥ 85% relative humidity. The filling volume per well was 1000 µL. The BioLector conducted a backscatter measurement every 10 min until the experiment was manually stopped.

### Lab-scale stirred tank bioreactor cultivation

*Aspergillus giganteus* was cultivated in a 1.5 L laboratory scale reactor (DASGIP, Eppendorf, Jülich/Germany) equipped with a pH sensor (405-DPAS-SC-K8S/225/120, Mettler-Toledo, OH) and a DO sensor (VisiFerm DO 225, Hamilton, Bonaduz, Switzerland) at 30 °C. The heat stable components of the medium were autoclaved in the reactor vessel. Afterwards the filtered components of the vitamin, trace element and salt stock solutions were added in a sterile manner before the DO sensors were calibrated. For the cultivation the filling volume was 700 mL and the pH was kept constant at 3.5 through addition of 2.5 M H_2_SO_4_ and 6 M KOH. The agitation and aeration rates were set to 400 rpm and 0.5 vvm, respectively. If required, antifoam (AF204, Sigma, St. Louis, MO) was added. The medium was inoculated with preserved spores to a final concentration of 1 × 10^6^ spores mL^−1^.

### Cell dry weight analysis

A total of 1000 µL culture suspension was added onto a pre-weighed spin down filter tube (cellulose-acetate membrane with a cut of size of 0.22 µm) (Corning^®^ Costar^®^ Spin-X, Sigma-Aldrich, St. Louis, Missouri/USA). The cells were separated via centrifugation at 13,000*g* for 5 min, the flow through (supernatant) transferred into a new 1.5 mL Eppendorf reaction tube and the cells washed with 500 µL of 0.9% (w v^−1^) NaCl. The washed filter tubes were subsequently dried at 80 C for 24 h, acclimatized to room temperature in an exsiccator for another 24 h and then weight on a precision scale.

### Morphology analysis

Morphology was analyzed via light microscope imaging. Pictures were taken with a Leica DMLB instrument equipped with a 4× objective (Leica Microsystems, Wetzlar, Germany) and a Thorlabs DCC1545M camera (Thorlabs, Newton, New Jersey, USA). Each image in the manuscript shows a size of 2.85 mm in width and 2.3 mm in height.

### Secretome analysis

The cell free supernatant was analyzed for secreted proteins. The NuPAGE^®^ kit was used to prepare the samples. 19.5 µL of the supernatant was added to 7.5 µL NuPAGE^®^ 4× sample buffer and 3 µL of NuPAGE^®^ 10× reducing agent. Afterwards the samples were boiled for 10 min. The protein separation was performed via SDS-PAGE on precast SDS-PAGE gels (15%) and visualized with Coomassie brilliant blue staining.

LC–MS/MS analysis was performed on a AB ScieX Triple ToF 6600 connected with an Agilent 1260 HPLC as described in literature [36] from the last sampling time points of the bioreactor cultivations with CaCl_2_. The secretome data is available at the PRIDE [37] repository with the identifier PXD013751. Each sample was injected three times as technical replicates.

### Statistical analysis

The relative mean coefficient of variation was calculated as an indicator for reproducibility. The arithmetic mean $\bar{x}_{i}$ of eight biological replicates and the corresponding standard deviation $s_{i}$ of each backscatter measurement was calculated for each measurement cycle i. The coefficient of variation $c_{v, i}$ was calculated according to Eq. 1.

1 $$c_{v, i} = \frac{{s_{i} }}{{\bar{x}_{i} }}$$

The sum of $c_{v, i}$ for all data points with an increase in scattered light measurement above the limit of detection (α = 0.01) (i = 1) until the end of cultivation (n) was divided by the number of cycles (m) to calculate relative mean coefficient of variation (Eq. 2).

2 $$rmc_{v} = \frac{{\mathop \sum \nolimits_{i = 1}^{i = n} c_{v,i} }}{m}$$

## Additional files

**Additional file 1.** Complex YEPD medium was inoculated with 2 × 10^6^ spores mL^−1^. *Aspergillus giganteus* was cultivated in a Flowerplate at 1100 rpm and 30 °C for 70 h. The biomass was analyzed non-invasively via scattered light measurement. A: Single well cultivations B: The mean (thick line) and standard deviation (lighter area) of eight biological replicates are shown. C: Microscopic images showing the different stages of morphology throughout the cultivation.

**Additional file 2.** Influence of shaking frequency variation and additives on the backscatter measurement with a Flowerplate. Complex YEPD medium was inoculated with 2 × 10^6^ spores mL^−1^. *Aspergillus giganteus* was cultivated in a Flowerplate at 30 °C for 70 h. The biomass was analyzed non-invasively via scattered light measurement. The mean (thick line) and standard deviation (lighter area) of eight biological replicates are shown.

**Additional file 3.** Influence of shaking frequency variation and additives on the backscatter measurement with a round well plate. Complex YEPD medium was inoculated with 2 × 10^6^ spores mL^−1^. *Aspergillus giganteus* was cultivated in a round well plate at 30 °C for 70 h. The biomass was analyzed non-invasively via scattered light measurement. The mean (thick line) and standard deviation (lighter area) of eight biological replicates are shown. Data for 850 rpm in the round well plate are shown in Fig. 3.

**Additional file 4.** Morphology analysis via microscopic imaging (40×) of bioreactor cultivation and BioLector cultivation. Each image shows a clipping of 2.85 mm in width and 2.3 mm in height. No clear difference in size can be seen between the two cultivation systems.

## Data Availability

The datasets used and/or analyzed during the current study are available from the corresponding author on reasonable request.

## Abbreviations

w v^−1^: weight per volume
MTP: microtiter plate
FP: flowerplate
RWP: round well plate
AFP: antifungal protein
YEPD: yeast extract peptone dextrose
rmc_v_: relative mean coefficient of variation
MPEC: microparticle enhanced cultivation
kDa: kilo Dalton
p-value: probability value
Na_4_EDTA: ethylenediaminetetraacetic acid sodium salt
SDS-PAGE: sodium dodecyl sulfate polyacrylamide gel electrophoresis
