# Supplementary figures and images for: A closer look at Aspergillus: online monitoring via scattered light enables reproducible phenotyping
Source: Fungal Biol Biotechnol. 2019 Aug 5;6:11. doi: 10.1186/s40694-019-0073-x (PMC6681481; doi:10.1186/s40694-019-0073-x)

A

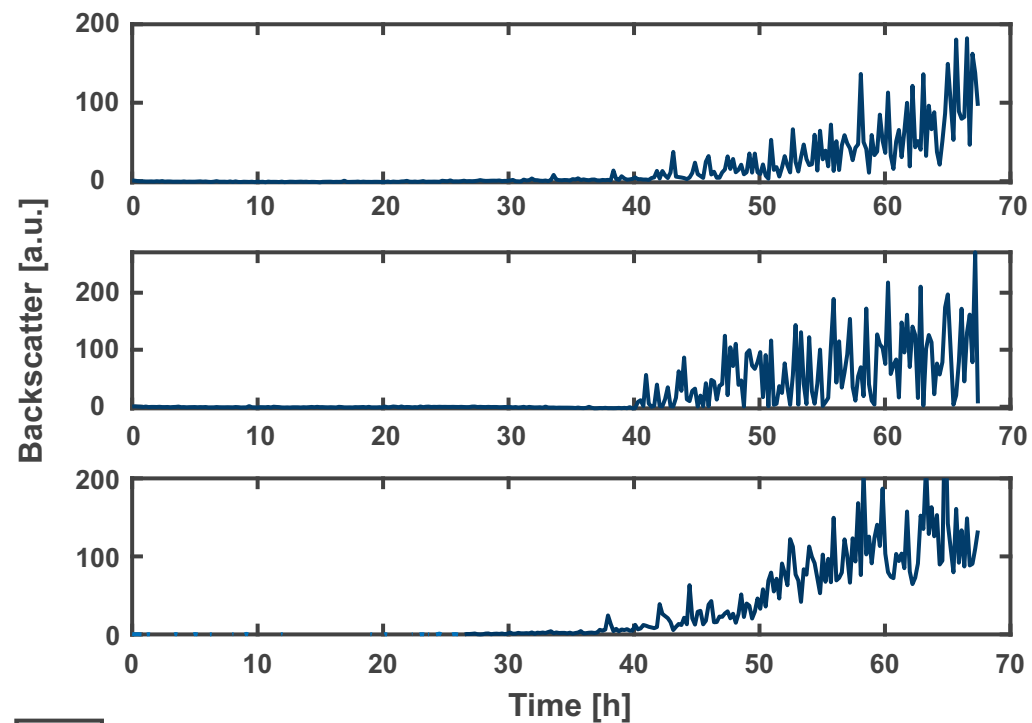

B

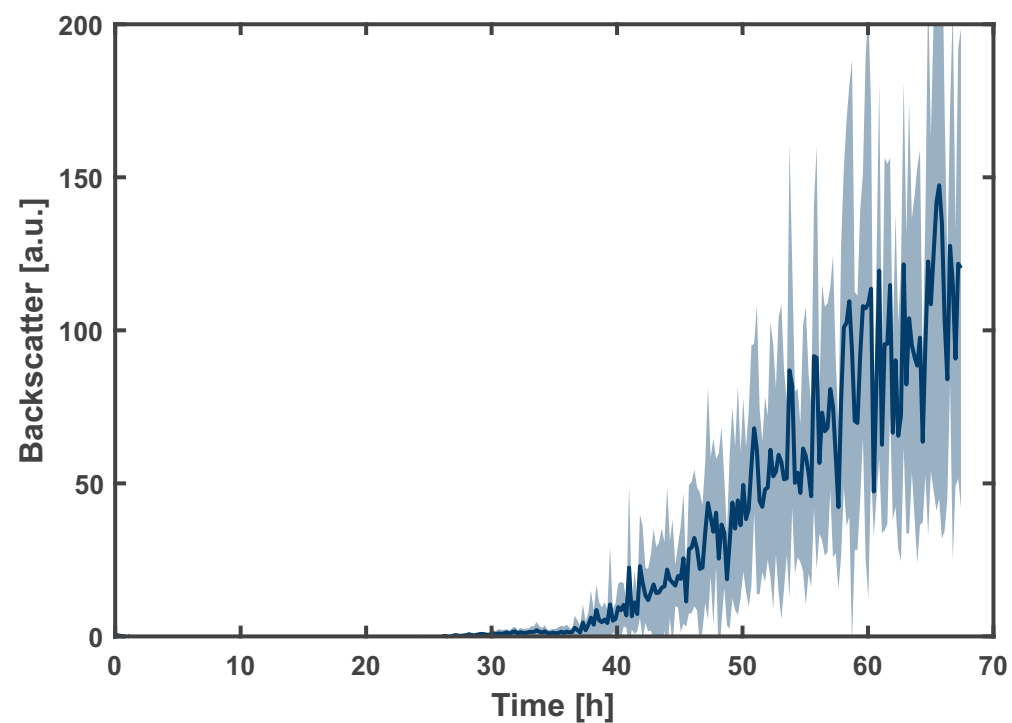

C

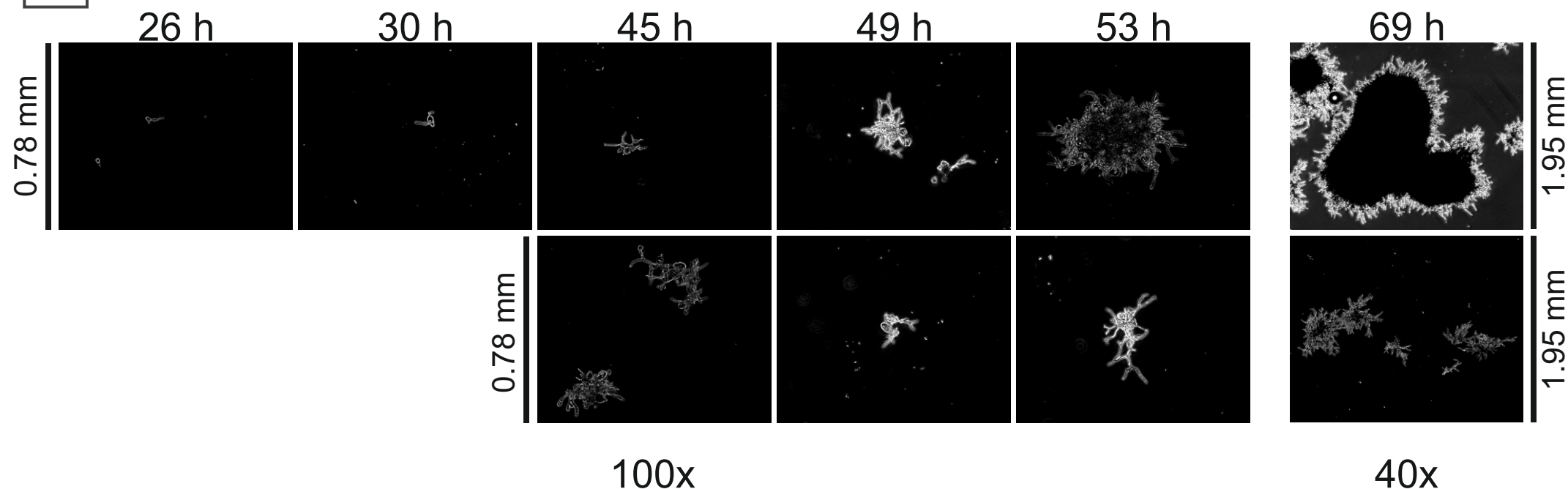

Supplement: Supplementary file 1 — Additional file 1. Complex YEPD medium was inoculated with 2 × 10^6^ spores mL−1. Aspergillus giganteus was cultivated in a Flowerplate at 1100 rpm and 30 °C for 70 h. The biomass was analyzed non-invasively via scattered light measurement. A: Single well cultivations B: The mean (thick line) and standard deviation (lighter area) of eight biological replicates are shown. C: Microscopic images showing the different stages of morphology throughout the cultivation. [file 40694_2019_73_MOESM1_ESM.pdf]

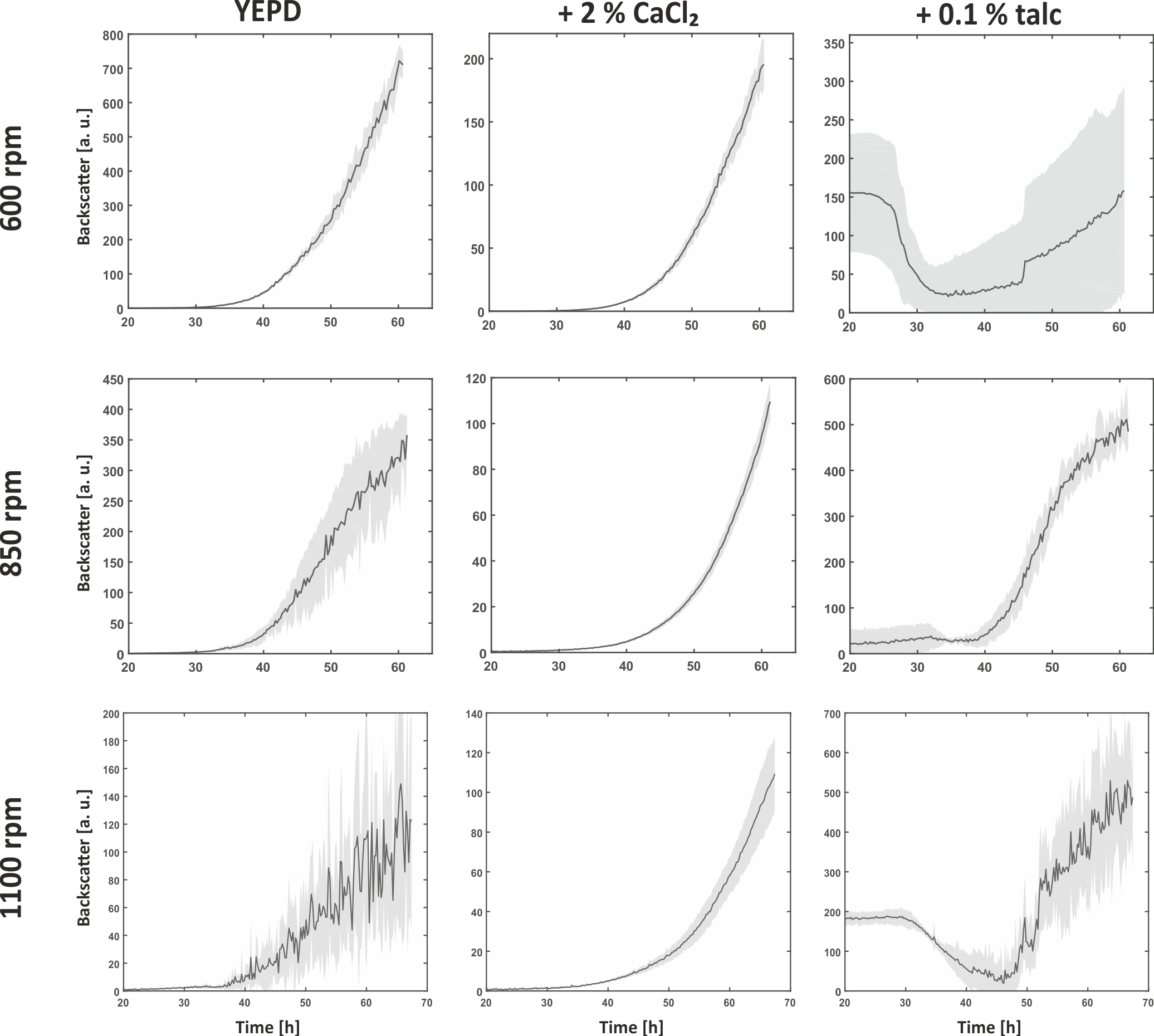

Supplement: Supplementary file 2 — Additional file 2. Influence of shaking frequency variation and additives on the backscatter measurement with a Flowerplate. Complex YEPD medium was inoculated with 2 × 10^6^ spores mL−1. Aspergillus giganteus was cultivated in a Flowerplate at 30 °C for 70 h. The biomass was analyzed non-invasively via scattered light measurement. The mean (thick line) and standard deviation (lighter area) of eight biological replicates are shown. [file 40694_2019_73_MOESM2_ESM.png]

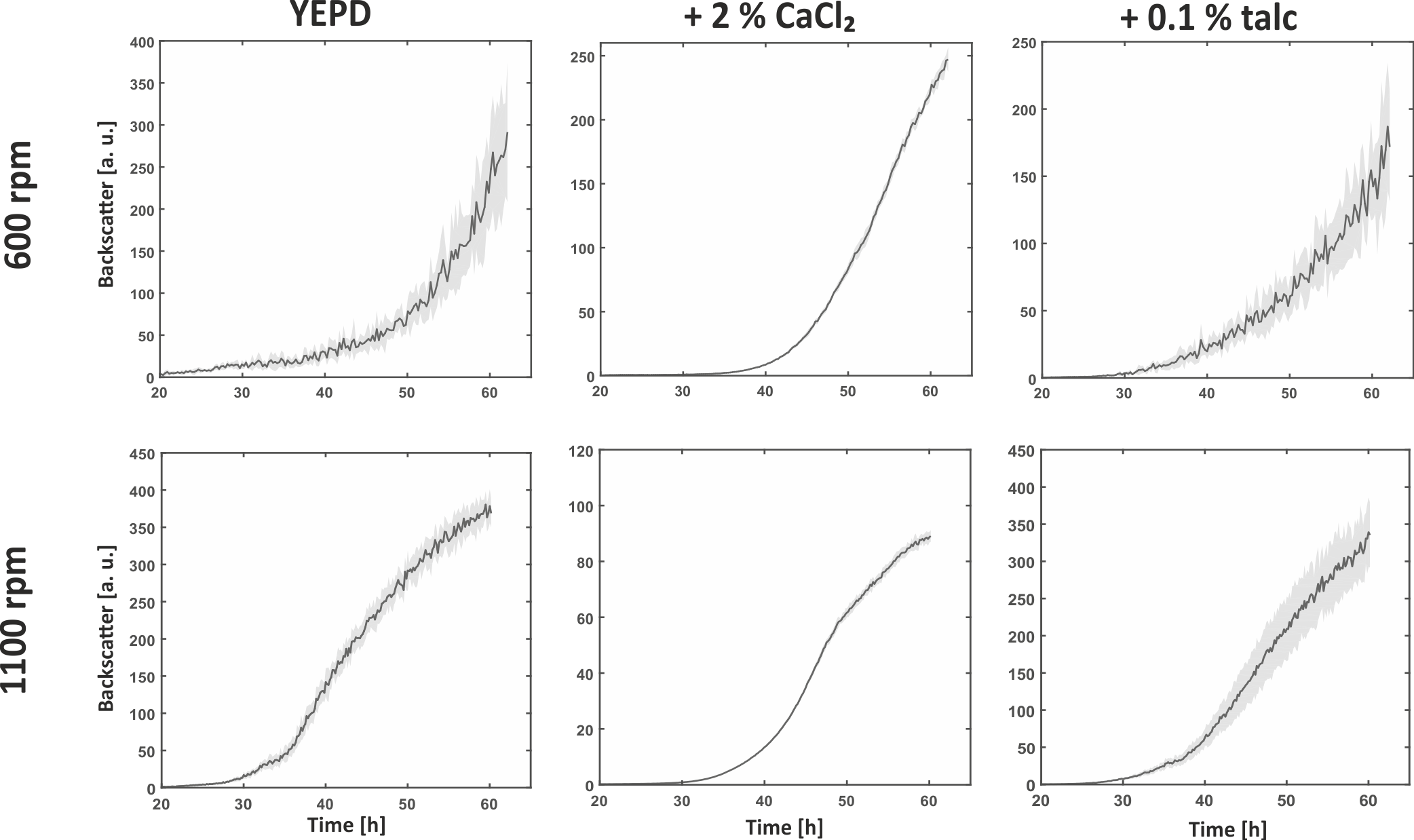

Supplement: Supplementary file 3 — Additional file 3. Influence of shaking frequency variation and additives on the backscatter measurement with a round well plate. Complex YEPD medium was inoculated with 2 × 10^6^ spores mL−1. Aspergillus giganteus was cultivated in a round well plate at 30 °C for 70 h. The biomass was analyzed non-invasively via scattered light measurement. The mean (thick line) and standard deviation (lighter area) of eight biological replicates are shown. Data for 850 rpm in the round well plate are shown in Fig. 3. [file 40694_2019_73_MOESM3_ESM.png]

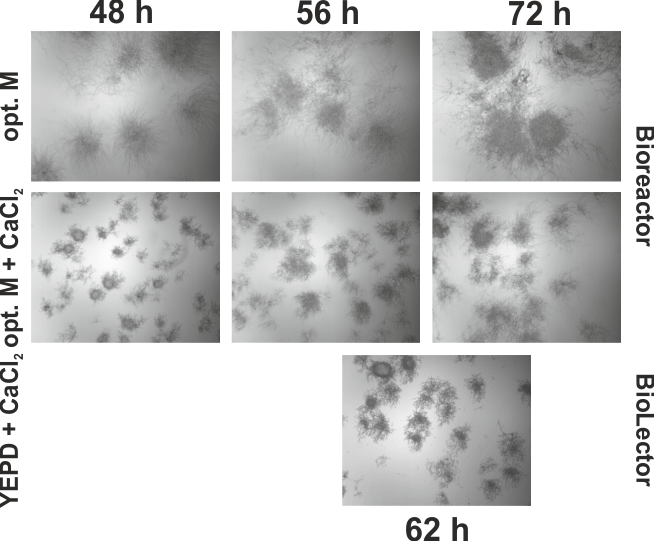

Supplement: Supplementary file 4 — Additional file 4. Morphology analysis via microscopic imaging (40×) of bioreactor cultivation and BioLector cultivation. Each image shows a clipping of 2.85 mm in width and 2.3 mm in height. No clear difference in size can be seen between the two cultivation systems. [file 40694_2019_73_MOESM4_ESM.png]
